# Recovery of post stroke proximal arm function, driven by complex neuroplastic bilateral brain activation patterns and predicted by baseline motor dysfunction severity

**DOI:** 10.3389/fnhum.2015.00394

**Published:** 2015-07-22

**Authors:** Svetlana Pundik, Jessica P. McCabe, Ken Hrovat, Alice Erica Fredrickson, Curtis Tatsuoka, I Jung Feng, Janis J. Daly

**Affiliations:** ^1^Department of Neurology, Case Western Reserve University School of MedicineCleveland, OH, USA; ^2^Neurology Service, Cleveland VA Medical CenterCleveland, OH, USA; ^3^Department of Epidemiology and Biostatistics, Case Western Reserve UniversityCleveland, OH, USA; ^4^Department of Neurology, College of Medicine, University of FloridaGainsville, FL, USA; ^5^North Florida/South Georgia, Gainesville VA Medical Center, Brain Rehabilitation Research CenterGainsville, FL, USA

**Keywords:** chronic stroke, motor rehabilitation, functional Magnetic Resonance Imaging, neuroplasticity, shoulder/elbow movement task, upper extremity motor function, motor recovery, motor learning

## Abstract

Objectives: Neuroplastic changes that drive recovery of shoulder/elbow function after stroke have been poorly understood. The purpose of this study was to determine the relationship between neuroplastic brain changes related to shoulder/elbow movement control in response to treatment and recovery of arm motor function in chronic stroke survivors.Methods: Twenty-three chronic stroke survivors were treated with 12 weeks of arm rehabilitation. Outcome measures included functional Magnetic Resonance Imaging (fMRI) for the shoulder/elbow components of reach and a skilled motor function test (Arm Motor Abilities Test, AMAT), collected before and after treatment.Results: We observed two patterns of neuroplastic changes that were associated with gains in motor function: decreased or increased task-related brain activation. Those with significantly better motor function at baseline exhibited a decrease in brain activation in response to treatment, evident in the ipsilesional primary motor and contralesional supplementary motor regions; in contrast, those with greater baseline motor impairment, exhibited increased brain activation in response to treatment. There was a linear relationship between greater functional gain (AMAT) and increased activation in bilateral primary motor, contralesional primary and secondary sensory regions, and contralesional lateral premotor area, after adjusting for baseline AMAT, age, and time since stroke.Conclusions: Recovery of functional reach involves recruitment of several contralesional and bilateral primary motor regions. In response to intensive therapy, the direction of functional brain change (i.e., increase or decrease in task-related brain recruitment) for shoulder/elbow reach components depends on baseline level of motor function and may represent either different phases of recovery or different patterns of neuroplasticity that drive functional recovery.

## Introduction

Motor deficits are life changing and devastating consequences of stroke. After 3–6 months following stroke, rehabilitation efforts decrease or cease altogether, despite remaining dysfunction in interpret many stroke survivors (Teasell et al., 2012). However, even years after stroke, an appropriately structured and sufficiently dosed rehabilitation program can produce statistically significant gains in motor function (Wolf et al., 2006; Lo et al., 2010; Whitall et al., 2011; Wu et al., 2013; McCabe et al., 2015). Unfortunately, in these studies not all subjects responded to the provided treatment, and participants did not recover normal function. In order to understand why some patients recover function better than others and to better guide the development of more specifically targeted rehabilitation methods, it is important to gain a more complete understanding of the mechanisms of the recovery of brain control of motor function.

To date, rehabilitation-related neuroplasticity research addressing functional brain changes has mostly focused on distal arm tasks and single joint elbow flexion/extension movements and information gained from functional imaging. And, these studies examined mostly mildly impaired individuals (Carey et al., 2002; Johansen-Berg et al., 2002; Dong et al., 2007; Takahashi et al., 2008; Boyd et al., 2010), and some studied moderately affected stroke survivors (Luft et al., 2004; Whitall et al., 2011). In contrast, neuroplastic brain changes in severely impaired individuals have been largely understudied (Page et al., 2010). Brain control of recovery of the shoulder/elbow reach task has not been studied with regard to neuroplastic mechanisms, although a couple of very important studies described neuroplastic changes related to elbow movement control (Luft et al., 2004; Whitall et al., 2011). There are studies of wrist/hand movements that have provided some insight into the relationship between rehabilitation-related neuroplasticity and recovery of wrist/hand related movements (Carey et al., 2002; Johansen-Berg et al., 2002; Schaechter et al., 2002; Ward et al., 2003; Takahashi et al., 2008; Page et al., 2010; Whitall et al., 2011; Kononen et al., 2012). However, it has been difficult interpret the literature on wrist/hand because of the mixed findings regarding whether motor improvement is driven by either an increase (Johansen-Berg et al., 2002; Dong et al., 2006; Schaechter et al., 2006; Takahashi et al., 2008; Page et al., 2010; Kononen et al., 2012) or a decrease of task-related brain activation (Wittenberg et al., 2003; Dong et al., 2007; Takahashi et al., 2008; Page et al., 2010; Kononen et al., 2012) driving recovery of wrist/hand function. To date, the differentiating factor for the mixed findings has been attributed to the degree of damage in the ipsilesional corticospinal tracts (Feydy et al., 2002; Hamzei et al., 2006); that is, for example, those with relatively preserved integrity of the corticospinal tract showed a decrease in brain activity or “focusing” of brain activity that was associated with recovery of motor control.

For functional recovery of proximal arm movement, there are existing reports on only isolated elbow movement and for more moderately impaired stroke survivors (e.g., mean Fugl-Meyer score, 32 points; Whitall et al., 2011); these few important studies provided some insight into the relationship between elbow movement gains and brain activation changes (Luft et al., 2004; Globas et al., 2011; Whitall et al., 2011; Stark et al., 2012).

No information is available regarding a direct relationship between brain pattern changes and recovery of the shoulder/elbow reach task. Yet, recovery of the functional reach movement (e.g., shoulder flexion/elbow extension components) is critical for positioning and stabilizing the limb for functional activities. Therefore, given the paucity of information regarding the brain changes driving recovery of shoulder/elbow reach movement components in severely impaired, chronic stroke survivors, our primary objective was to identify, for this group, the patterns of brain activation change that drive recovery of the multi-joint shoulder/elbow reach task in response to training. A secondary objective was to characterize the relationship between the degree of functional motor gain and the extent of brain activation change, in response to treatment.

## Materials and Methods

### Subjects

We enrolled 23 individuals with chronic stroke (stroke onset >6 months). The main inclusion criteria were as follows: age >21 years; single stroke; ≥ a trace muscle contraction for the wrist extensors and elbow and shoulder major muscle groups in the stroke-affected arm; and no contraindications for Magnetic Resonance Imaging (MRI). Eleven healthy control subjects were enrolled for functional Magnetic Resonance Imaging (fMRI) testing. Control subjects’ data was used for fMRI data analysis as described below. The study protocol was approved by the Institutional Review Board in the Medical Center; informed written consent was obtained for each subject.

### Intervention

**Figure 1:**
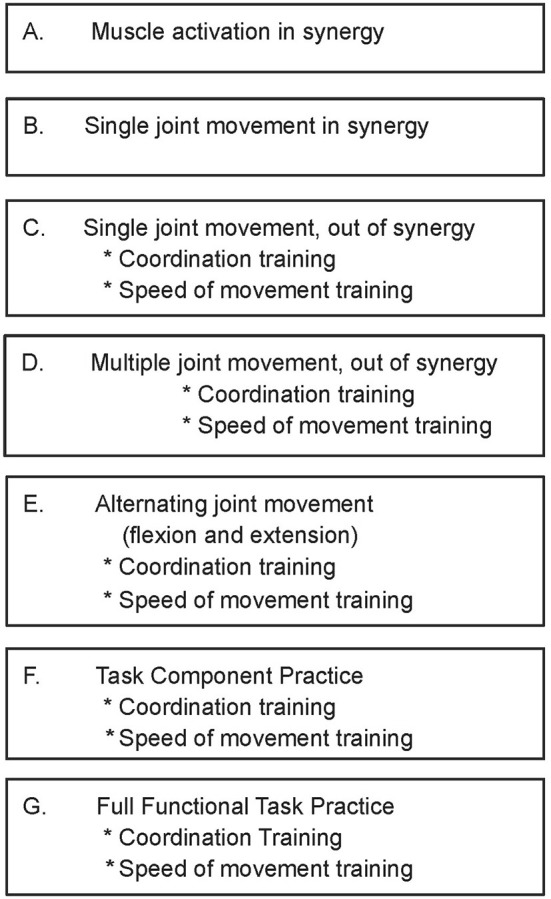
**Upper limb training protocol: treatment progression hierarchy for coordinated movement practice**. Reproduced with permission of Arch PMR, McCabe et al. (2015).

Upper limb motor therapy was provided by licensed therapists 5 h/day, 5 days per week for 12 weeks (total of 60 visits). Treatment was based on the following established principles of motor learning: high repetition of everyday functional tasks or task components(Pascual-Leone and Torres, 1993; Butefisch et al., 1995; Elbert et al., 1995; Dean and Shepherd, 1997), part- vs. whole-task practice (Schmidt, 1991; Shumway-Cook and Woollacott, 2007), practice of movement as close to normal as possible (Nudo et al., 1996a,b), generalization of newly gained movement capability to novel tasks (Shumway-Cook and Woollacott, 2007), and attention to task (Singer et al., 1993). In addition to these principles and based on prior work (Daly et al., 2005; McCabe et al., 2015), we utilized a motor task difficulty hierarchy to guide progression of treatment (Figure 1) toward normal movement. In a given daily treatment session, subjects practiced an array of upper extremity functional tasks that incorporated the reach components of shoulder flexion and elbow extension. Examples of functional tasks included picking up a tray and placing it on a counter, reaching into a cupboard for a can, eating with utensils, and opening/closing a door. Subjects practiced a given functional task at a level that was challenging. In order to determine the appropriate level of challenge, the therapist first analyzed the subject’s performance capability for a given task. If the subject could not successfully perform the whole task, the task was decomposed into component parts and the subject practiced the task components. Practice-assistance was provided early in the training, and the subject was progressed to independent practice, as volitional control was gained. As task component performance improved, the subject then practiced the whole task. A variety of upper limb functional tasks were practiced by subjects throughout the intervention protocol.

### Motor Function Outcome Measure

The Arm Motor Ability Test (AMAT) was administered before and after the rehabilitation intervention. The AMAT consists of 13 complex upper limb functional tasks of activities of daily living that incorporate shoulder flexion/elbow extension. The AMAT is measured according to the time to complete the task. It has high inter-rater reliability(Spearman correlations = 0.97–0.99), internal consistency, sensitivity to change, satisfactory concurrent validity (Kopp et al., 1997) and has been identified in a recent systematic review as a valid and reliable instrument for arm-hand assessment at the level of The International Classification of Functioning, Disability and Health (ICF) activity for individuals with stroke (Lemmens et al., 2012). The worst possible score is 2940 s indicating inability to perform any portion of any of the 13 tasks, and average healthy adult score is 426 ± 120 s (Rinehart et al., 2009).

### fMRI Data Acquisition

MRI was acquired using a Siemens Symphony 1.5 T system with a circularly polarized head coil and an interleaved multi-slice gradient-echo echoplanar imaging (EPI) sequence. Blood-oxygenation level dependent (BOLD) images were obtained with in-plane resolution of 3 × 3 mm, repetition time (TR) = 3.87 s, echo time (TE) = 50 ms, flip angle = 90°, and 36 axial slices through the entire brain. For axial T1 images, in-plane resolution was 1 × 1 mm, TR = 2.16 s, TE = 3.45 ms, and flip angle was 15°.

**Figure 2:**
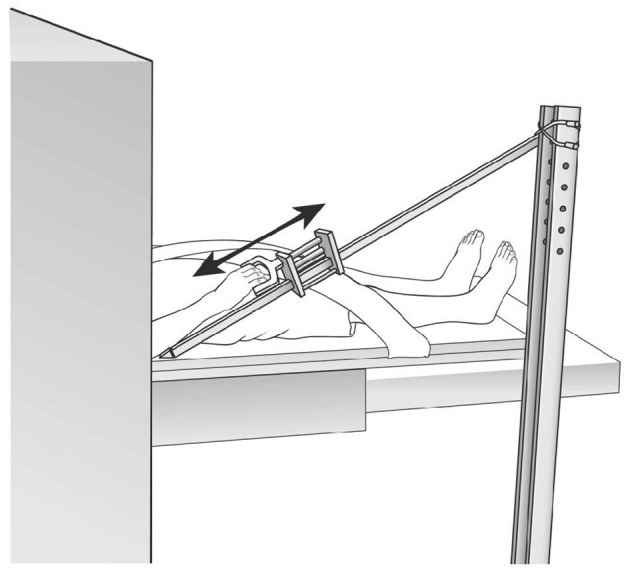
**functional Magnetic Resonance Imaging (fMRI) set up**. Reproduced with permission of J. Neurosci. Methods, Daly et al. (2008).

The fMRI protocol was a block design with alternating move and rest blocks (10 scans per block; 40 s per block); rest-move cycles were repeated five times. The shoulder/elbow reach motor task for the paretic arm was performed by flexing the shoulder/extending the elbow, sliding the arm along a wooden guide placed at a 30° angle with respect to the horizontal bed, and with the hand secured in a handle (Figure 2). The resting position was with shoulder resting on the MRI bed and elbow at a 30° angle from horizontal, with forearm supported by the wooden movement guide. Audio cues to begin the motor task and to rest were delivered through headphones. The movements were performed in a slow, continuous manner, at a rate of 0.2 Hz. We monitored for undesired mirror movement in the uninvolved, non-tested arm (anterior deltoid, triceps, biceps, wrist and finger flexors and extensors), using an MRI-compatible electromyographical (EMG) system (BrainVision LLC, Morrisville, NC, USA; Daly et al., 2008). EMG data from the uninvolved arm were acquired during fMRI data acquisition and subsequently analyzed for the presence of undesired muscle activation in the non-tested arm; EMG amplitude greater than 2 SD of the resting EMG was the criterion that determined whether a given muscle was activated (Bogey et al., 1992; Levin and Dimov, 1997). Scans were discarded in the event that they were associated with an inter-scan interval containing EMG signal that exhibited the “on” condition for the muscles in the uninvolved, non-tested arm (Daly et al., 2008).

There were two practice sessions, as follows: one, the day before the actual test session (outside the MRI department) and second, on the testing day (in the MRI suite). The goals of the practice sessions were to insure that these criteria were achieved: (1) movement was isolated to the tested arm and no other movements of the body or the uninvolved arm were observed; (2) the speed of movement was kept within the speed of 0.2 Hz. Using these practice methods, as well as crisscrossed torso strapping, and custom head stabilization materials within the headrest, we were able to maintain head movement during scanning to <0.4 mm of translation in the x, y, and z directions and <0.4° of rotation.

### fMRI Data Analysis

MRI data were processed and analyzed using the Statistical Parametric Mapping (SPM5) package (Wellcome Department of Imaging Neuroscience at University College London, UK), along with custom in-house software analysis packages designed by our lab using the MATLAB (The MathWorks, Inc., Natick, MA, USA) technical computing environment.

According to standard methods, the fMRI data analysis preprocessing steps included slice-timing, head motion corrections, co-registration of anatomical and BOLD images, and brain parenchyma segmentation. Data were normalized to a standard template based on the Montreal Neurological Institute (MNI) reference data. After the standard procedure of transformation into MNI space, images were inspected by a neurologist/neuroscientist in order to confirm that the transformation was appropriate and proportional for all brain regions, including the region containing the lesion, resulting in successful registration for all subjects. Spatial smoothing was performed with a 6 mm^3^, full-width at half maximum Gaussian kernel. For the analyses, images were right/left flipped in order to align the lesion hemispheres which were all contralateral to the tested arm (Crafton et al., 2003; Loubinoux et al., 2007; Nair et al., 2007).

fMRI activation maps for each subject/session were determined by contrasting rest vs. move data using voxel-based *t*-test analysis. Activation threshold was determined using a group-wise analysis based on the aggregate data from baseline fMRI sessions for stroke subjects and 11 control subjects. For control subjects, we tested the dominant arm according to the procedures used for stroke subjects. For determining activation threshold, we contrasted rest and move scans using an independent sample *t*-test (*p* < 0.05), with standard correction for multiple comparisons (Benjamini and Hochberg, 1995; Genovese et al., 2002). The resultant threshold was *p* = 0.00062.

Regions of interest (ROIs) were identified using the standard SPM procedure (Dong et al., 2006; Nair et al., 2007; Page et al., 2010; Whitall et al., 2011), and according to Brodmann areas (BAs), and after which an inspection was conducted to ensure proper registration. An inspection of activation maps was performed; there was no spurious activation present in the lesion cavities. Regional fMRI activation was expressed as active voxel count in a given ROI extracted from whole brain activation maps. The primary ROI was the ipsilesional primary M1 (BA 4). Secondary exploratory analyses were conducted for the contralesional M1 region; and the following bilateral ROIs: primary somatosensory region (S1; BA 1, 2, 3); lateral premotor area (LPM; lateral surface of BA 6; Picard and Strick, 2001); supplementary motor area (SMA) proper (medial portion BA 6 that is posterior to the anterior commissure line; Picard and Strick, 1996); secondary sensory region (SII; BA 5, 7); and associative sensory (AS; BA 39, 40) region.

### Statistical Analysis

Statistical analysis was performed using R statistical software (version 2.14.1) and SPSS (v.22, IBM Statistics). Graphical inspection of histograms and Shapiro-Wilks tests were performed to check normality assumptions; nonparametric statistics were used for non-normally distributed data. A *p* ≤ 0.05 indicated statistical significance, and we used Holm-Bonferroni correction (Holm, 1979) for multiple comparisons. We conducted several secondary analyses. We used multiple linear regression analysis to study the relationship between the gain in motor function and change in brain activation; covariates in the model were baseline AMAT, age, and time since stroke. We calculated the correlation between baseline AMAT score and AMAT change score using the Spearman correlation method. We calculated percent gain for the AMAT, relative to baseline. We generated AMAT descriptive statistics baseline and post-treatment AMAT mean and standard deviation. After analyzing data *according to each ROI*, we found that some subjects who showed high consistency across ROI’s with regard to either an increase or a decrease of brain activation in response to treatment; and some subjects exhibited a mix of increase or decrease of activation across their respective ROI’s. Therefore, we categorized subjects into *three groups according to their consistency or lack thereof across their own ROI’s, as follows*: (1) subjects who increased activation across at least 11 out of 12 ROIs; (2) subjects whose activation across at least 11 out of 12 ROIs decreased or stayed the same; and (3) subjects with mixed patterns across ROIs. We generated descriptive statistics for the mixed group 3. And we used the Kruskal-Wallis three-way comparison to compare the three groups, according to baseline AMAT and according to AMAT gain score. To elucidate a three-way finding of statistical significance, we made *post hoc* pair-wise comparisons using the Mann-Whitney test.

## Results

### Subjects’ Characteristics

**Figure 3:**
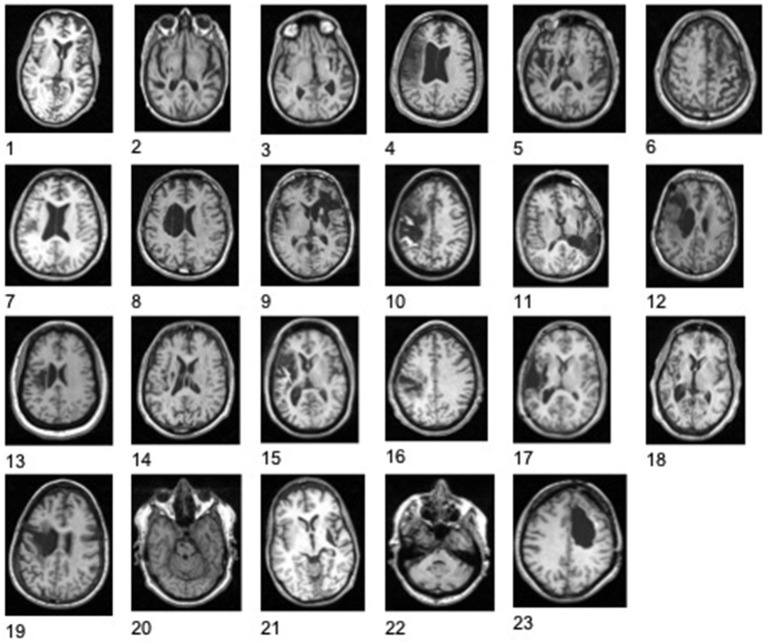
**Illustrations of stroke lessions**. Right side of each image represents right hemisphere.

Stroke subject characteristics are shown in Table 1. The subjects had moderate to severe deficits of upper extremity function with mean baseline Upper Extremity FM = 22.2 ± 8.6 (range 8–44; Duncan et al., 1992). Control subjects were 54.4 ± 12.9 years of age; 54% were female. Figure 3 shows the stroke lesion for each subject.

**Table 1:** **Subject characteristics**.

	Stroke subjects *n* = 23
Age in years, mean (std dev)	56.3 (12.8)
Female (%)	41
Stroke hemisphere (% Left)	55%
Stroke Type (% ischemic)	88.6%
Years since stroke	1.8 (1.1)
Lesion location n (%)
BG/IC	7 (30%)
Pons	2 (8.6%)
Frontal lobe	1 (2.3%)
Frontal/parietal lobes	3 (13%)
Frontal lobe/BG/IC	3 (13%)
Frontal/parietal lobes/BG/IC	5 (21.7%)
Frontal/parietal/temporal lobes/BG/IC	2 (8.6%)
Medical history
DM	17.4%
HTN	52.2%
Heart disease	21.7%
Smoking	56.5%

*Key: BG - basal ganglia, IC - internal capsule, DM - diabetes mellitus, HTN - hypertension*.

### Shoulder-Elbow Reach Task-Related fMRI Activation

**Figure 4:**
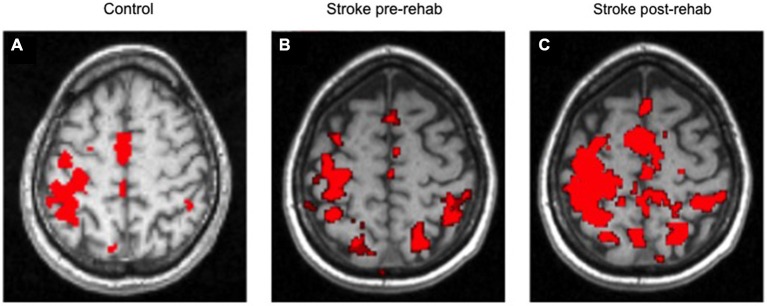
**Average control brain activation map (A); Example of stroke at Pre- Treatment (B) and Post Treatment (C)**. Left side of each image is contralateral to the moving arm.

Figure 4 shows the average control fMRI activation map and an example of the brain activation for a stroke survivor, both performing the shoulder flexion/elbow extension component of the reach task. In this example, there was an increase of brain activation at post treatment.

### Functional Gain Following Rehabilitation

Following rehabilitation, there was a statistically significant improvement of skilled motor function according to AMAT (pre-treatment, 1654 ± 658 s; post-treatment, 1253 ± 637 s, *p* < 0.0001). The post-treatment AMAT gain, expressed as percent of baseline score was 26 ± 21.2%. All (except one subject) demonstrated functional motor gains according to AMAT; the mean of AMAT gain was 419.5 ± 375 s. There was a very poor and non-significant correlation between baseline AMAT score and AMAT change score after therapy (*r* = 0.34, *p* = 0.2).

### Analysis by Single ROI: Less Motor Impairment at Baseline for Neuroplastic Pattern 2 Group (Those Who Decreased Brain Activation in Response to Treatment, for Each Given ROI) vs. Neuroplastic Pattern 1 Group (Increased Activation at Post-Treatment for Each Given ROI)

Following rehabilitation, some subjects decreased (Neuroplastic Pattern 2 group) and some increased or did not change in task-related regional brain activation (Neuroplastic Pattern 1 group), while all subjects in both groups, except one, improved their motor function. For each ROI, Table 2 provides a comparison of the Neuroplastic Pattern 1 Group (column 1) vs. the Neuroplastic Pattern 2 Group (column 2) with regard to their baseline motor function (AMAT scores, columns C vs. E). We found that those with significantly better motor function at baseline exhibited a decrease in brain activation in response to treatment (Neuroplastic Pattern 2 group), as evidenced in the ipsilesional primary motor region (primary measure, *p* = 0.01) and contralesional SMA (secondary measure, *p* = 0.004; Table 2, first row and last row, respectively; last column). For both Neuroplastic Pattern groups, there was improvement in motor function (AMAT score; *p* < 0.05).

**Table 2:** **For each ROI, comparison of two neuroplastic patterns, according to baseline motor function (AMAT)**.

		Neuroplastic Pattern 1 (increased fMRI activation after treatment)		Neuroplastic Pattern 2 (decreased or unchanged fMRI activation after treatment)	
	ROI	Sample size	AMAT (s) mean (SD)	Sample size	AMAT (s) mean (SD)	Pattern comparison *p* value
**Ipsilesional**	M1	14	1927.4 (429)	9	1230.8 (749)	0.01*
	AS	8	1753.7 (418)	15	1602.0 (765)	ns
	S1	13	1914.9 (444)	10	1316.6 (756)	0.03
	SII	11	1866.0 (484)	12	1461.2 (754)	ns
	LPM	11	1919.3 (462)	12	1412.3 (734)	ns
	SMA	11	1928.7 (466)	12	1403.7 (725)	0.05
**Contralesional**	M1	11	1889.9 (492)	12	1439.3 (736)	ns
	AS	7	1754.4 (448)	16	1611.2 (741)	ns
	S1	10	1933.0 (491)	13	1440.8 (707)	ns
	SII	9	1754.4 (438)	14	1590.8 (777)	ns
	LPM	10	1897.4 (461)	13	1468.1 (740)	ns
	SMA	11	2043.3 (347)	12	1298.6 (684)	0.004*

Key: *Significant p value after adjustment for multiple comparisons, according to Holm-Bonferroni method; ns, unadjusted p > 0.05; M1, primary motor; SMA, supplementary motor area; LPM, lateral premotor region; S1, primary somatosensory area; SII, secondary sensory region; AS, associative sensory; ROI, region of interest; AMAT, Arm Motor Abilities Test.

### Analysis by Single ROI: Neuroplastic Pattern 1 Group (Increased Post-treatment Activation for each Given ROI) had a Significantly Greater Gain in Complex Motor Function Tasks (AMAT) vs. Neuroplastic Pattern 2 Group (Decreased/unchanged Post-treatment activation)

Table 3 shows the comparison of the Neuroplastic Pattern Group1 vs. Group 2, according to gain on AMAT score for each ROI. For the primary ROI, ipsilesional M1, there was a statistically significantly greater gain in motor function for Pattern 1 (increase in brain activation in response to treatment) vs. for Pattern 2 (column F, row 1, Table 3). In our secondary analysis, only the contralesional AS region showed a significant difference in the AMAT score, with greater gain at post-treatment for Neuroplastic Pattern1 group. Though not maintaining statistical significance after correction for multiple comparisons, there was a trend in the same direction for ipsilesional somatosensory (SS), LPM; and contralesional SS, SII, LPM (Table 3, column F).

**Table 3:** **For each ROI, comparison of two neuroplastic patterns, according to change in AMAT score in response to rehabilitation**.

		Neuroplastic Pattern 1 (increased fMRI activation after treatment)		Neuroplastic Pattern 2 (decreased or unchanged fMRI activation after treatment)	
	ROI	Sample size	Baseline AMAT (sec)mean (SD)	Sample size	Baseline AMAT (sec)mean (SD)	Pattern comparison *p* value
**Ipsilesional**	M1	14	538.0 (386)	9	235.1 (282)	0.028*
	AS	8	549.5 (484)	15	350.1 (299)	ns
	S1	13	556.5 (395)	10	241.3 (274)	0.015
	SII	11	485.4 (305)	12	359.0 (434)	ns
	LPM	11	576.1 (422)	12	275.9 (271)	0.023
	SMA	11	547.9 (445)	12	301.7 (266)	ns
**Contralesional**	M1	11	612.3 (428)	12	242.8 (210)	0.023
	AS	7	715.9 (433)	16	289.8 (270)	0.004*
	S1	10	593.7 (441)	13	285.4 (261)	0.042
	SII	9	609.0 (438)	14	297.6 (283)	0.028
	LPM	10	595.8 (440)	13	283.8 (260)	0.049
	SMA	11	564.9 (444)	12	286.2 (250)	ns

Key: M1, primary motor; SMA, supplementary motor area; LPM, lateral premotor region; S1, primary somatosensory area; SII, secondary sensory region; AS, associative sensory; *significant p value after adjustment for multiple comparisons, according to Holm-Bonferonni method; ns, non-significant; ROI, region of interest.

### Analysis by Subject. Consistency of Brain Activation Change across ROIs in Response to Treatment: Comparison of Three Subject Groups: (1) Brain Activation Change of Consistent Increase vs. (2) Decrease vs. (3) Mixed Change in Response to Treatment

After analyzing data *according to each ROI*, it became apparent that some of the subjects were not uniform across their respective ROIs; therefore, we categorized subjects into three groups according to their consistency or lack thereof across their own ROI’s, as follows: (1) subjects who increased activation across at least 11 out 12 ROIs; (2) subjects whose activation across at least 11 out of 12 ROIs decreased or stayed the same; (3) subjects with mixed patterns across ROIs. Within the mixed group, the percent of ROIs with decreased activation was 50 ± 20% (mean ± SD), range: 25–83%.

In our three-way comparison for subjects who had a uniform pattern of change of activation across ROIs: (1) increased vs. (2) decreased or no changed; and (3) those with a mixed pattern of change in activation across the ROIs, we found significant difference for the three-way comparison according to AMAT baseline and AMAT gain score (Table 4). The *post hoc* pair-wise comparisons showed that those with consistent decreases across ROIs had significantly less baseline dysfunction (*p* = 0.02) than those with mixed brain changes across ROI’s (*p* = 0.02; Table 4 column 2 vs. column 3); and there were no other pair-wise significant comparisons for baseline AMAT. Pair-wise comparisons for AMAT gain showed that those with consistent increases had significantly greater AMAT gain vs. those with consistent decreases (*p* = 0.007; Table 4, column 1 vs. column 2); and there were no other pair-wise significant comparisons for AMAT gain score. The Neuroplastic Pattern groups were compared regarding age and time since stroke; and there were no significant differences (*p* > 0.05).

**Table 4:** **Comparison of three neuroplastic patterns (consistency or lack thereof across ROIs), according to baseline AMAT, AMAT gain, time since stroke and age**.

	Consistent** increased brain activation across ROI’s after treatment (*n* = 6)	Consistent** decrease (or unchanged) brain activation across ROIs after treatment (*n* = 7)	Mixed neuroplastic pattern across ROIs after treatment (*n* = 10)	*p* value*
AMAT at baseline (s), mean (SD)	1691.6(479)	1070.5(753)	2041.7(349)	0.04
AMAT improvement (s), mean (SD)	693.3(471)	169.8 (203)	430(309)	0.02
Age in years, mean(SD)	58.17(6.8)	57.57 (13.6)	51.7(14.6)	ns
Time since stroke, in years, mean(SD)	2.28(1.5)	1.99 (1.3)	1.34(0.4)	ns

Key: *p value for Kruskal-Wallis test comparing the three neuroplastic pattern changes after treatment. **Uniform pattern across all ROIs was considered if at least 11 out of 12 ROIs had the same direction of change in activation.

### Relationship Between Regional Brain Activation and Functional Motor Gain (AMAT)

**Figure 5:**
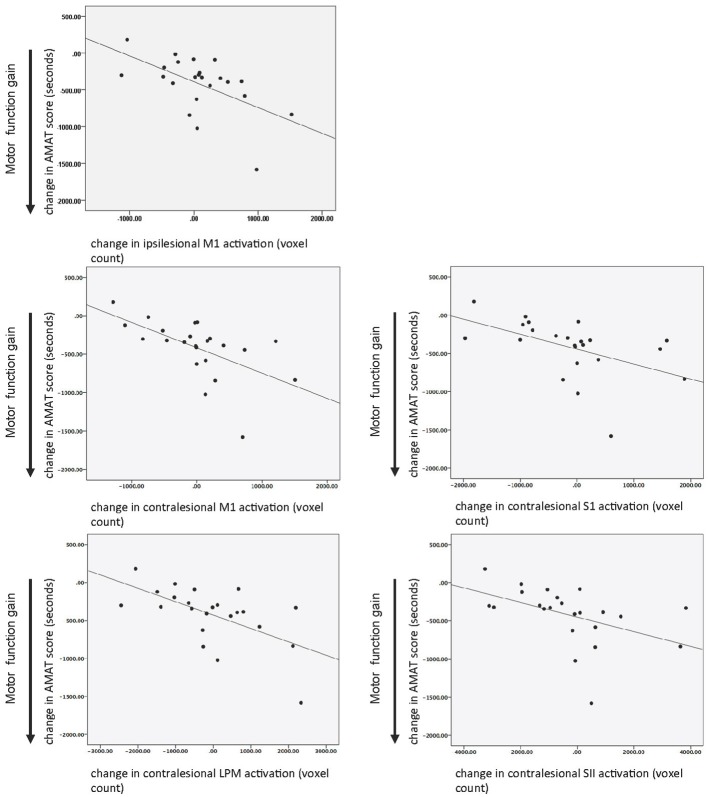
**Relationship between motor function gain (AMAT score) and change in task-related brain activation in response to treatment**. Key: M1, primary motor; LPM, lateral premotor region; S1, primary somatosensory area; SII, secondary sensory region; AMAT, Arm Motor Abilities Test.

There was a statistically significant relationship between AMAT gain score and change in fMRI activation for ipsilesional M1 and in contralesional M1, S1, SII, and LPM, while adjusting for baseline motor function, age, and time since stroke (Table 5). Figure 5 shows this relationship, for the ROIs that demonstrated a statistically significant relationship.

**Table 5:** **Regression analysis demonstrating a relationship between change in task-related regional brain activation and gain in skilled motor function (AMAT gain), adjusted for baseline AMAT, age and time since stroke**.

	ROIs	Parameter estimate for motor function gain (AMAT, s), *p* value	Parameter estimate for baseline AMAT (s), *p* value	Parameter estimate for time since stroke (years), *p* value	Parameter estimate for age (years), *p* value
**Ipsi**.	**M1**	0.77	0.19	−51.8	4.5
		*p* = 0.03*	ns	ns	ns
**Contralesional**	**M1**	0.87	0.28	169	2.8
		*p* = 0.015*	ns	ns	ns
	**LPM**	1.69	0.58	277	3.7
		*p* = 0.014*	ns	ns	ns
	**S1**	1.08	0.33	264	1.0
		*p* = 0.049*	ns	ns	ns
	**SII**	2.04	0.4	769	4.0
		*p* = 0.038*	ns	*p* = 0.02*	ns

Listed here are parameter estimates for the model variables (and p values). Key: *Significant at p ≤ 0.05. ns, non-significant (*p* > 0.05); ipsi, ipsilesional; M1, primary motor; SMA, supplementary motor area; LPM, premotor regions; S1, primary somatosensory area; SII, secondary sensory region; AMAT, Arm Motor Ability Test.

## Discussion

There are several study findings that extend the literature. First, to our knowledge, this is the first study to demonstrate for the proximal arm, shoulder/elbow reach components, a post-rehabilitation change in task-related brain activation. Second, two different neuroplastic patterns were observed in recovery of motor function following rehabilitation of chronic upper extremity motor deficits; the two different patterns of brain activation change were as follows: (1) an increase in volume of brain activation; or (2) a more focused (decreased) volume of activation. Some subjects had a more uniform pattern (ROI’s increased or ROI’s decreased) and others had a mixed pattern of neuroplastic change across ROIs. Third, baseline level of impairment was the single most important predictor of the type of neuroplastic change (i.e., increase or decrease in brain activation in response to treatment) driving recovery of arm function. Fourth, in response to rehabilitation, greater extent of gain in motor function was exhibited by those with more impairment at baseline; and notably, for those subjects, there was greater post-treatment increase in the volume of task-related brain activation, especially in the contralesional ROIs.

### Two Different Neuroplastic Patterns of Brain Change in Response to Treatment and Recovery of Motor Function, i.e., Increase or Decrease in Regional Brain Activations

These results for our study of the shoulder/elbow reach task are consistent with that of other separate studies of distal limb tasks, in which scientists reported contrasting findings of either an increase or a decrease in activation in response to treatment and recovery of motor function (Schaechter et al., 2002; Ward et al., 2003; Sasaki et al., 2012); these prior published findings that pitted studies against each other fueled an “either/or” debate that may be too narrowly and simplistically framed. Insofar as the current shoulder/elbow data are relevant to the wrist/hand data, our findings demonstrate one of the reasons for the contrasting results in these prior studies. That is, we demonstrated that the direction of brain change (increase or decrease in activation) was associated with level of motor dysfunction at baseline; those who showed reduced activation at post-treatment were less disabled at baseline.

Still others, described that the direction of neuroplastic response was dependent on the extent of ipsilesional corticospinal tract damage (Feydy et al., 2002; Hamzei et al., 2006). These important studies(Feydy et al., 2002; Hamzei et al., 2006) however, were unable to show a relationship between the direction of neuroplastic changes and amount of functional gain. In contrast, our results showed that the relationship was more complex than anticipated; that is, those with reduction in brain activation (more focused) in response to training, had a smaller gain score for motor function compared with those exhibiting more brain activation in response to training (greater gain score for them). There may be a number of reasons why we were able to achieve these results compared to other reported studies. Differences with other studies are as follows: (1) we studied the proximal shoulder/elbow reach task; (2) study of chronic and more severely involved stroke survivors; and (3) the intensity of the treatment received (300 h of therapy).

Neuroplastic brain changes during recovery in chronic stroke have been studied using a number of methods in addition to fMRI. This work has shown that recovery of motor function can involve multimodal structural and functional re-organization (reviewed in Di Pino et al., 2014). The complexity of post-stroke reorganization is evident from studies describing bimodal synaptic plasticity, as well as decrease of inhibitory and increase of excitatory neurotransmission (Clarkson et al., 2010; Carmichael, 2012). Successful recovery in the chronic stage can involve recruitment of alternative single or multiple functional brain regions in both ipsilesional and contralesional hemispheres (Swayne et al., 2008; Di Pino et al., 2014). This complexity is likely underlying the current results of contrasting brain changes associated with baseline severity level, as well as the complexity of brain changes within a given subject across their ROI’s (Table 4). Given the breadth and complexity of the potential neuroplastic mechanisms driving motor recovery, it is reasonable to consider that there are differing pathways of the neuroplastic changes that are driving recovery in severely vs. mildly impaired stroke survivors, as well as differing pathways within one subject across their ROI’s.

### Influence of Baseline Impairment Level on Engagement of the Type of Change in Brain Activation in Response to Treatment

Baseline characteristics that predict patterns of brain reorganization have been investigated by others, but mostly for wrist/hand movement, and only for unidirectional change in brain activation. First, baseline impairment was identified as a predictive factor by Kononen et al. (2012) who observed in a study of constraint-induced therapy that the presence of more severe impairment at baseline correlated with greater increase in ipsilesional M1 activation, in response to treatment.

In the current study of the shoulder/elbow reach task, our results in the M1 region are consistent with their findings for wrist/hand, in that those who employed increased activation in response to treatment (Neuroplastic Pattern 1, Table 2) had greater baseline functional deficit than those who employed a decrease in activation in response to treatment. The second reported predictor is better-preserved ipsilateral tracts, which were reported to correlate with a decrease (more focused) in brain activation after treatment, according to motor evoked potentials (MEPs) and diffusion imaging tractography (Feydy et al., 2002; Hamzei et al., 2006). Third, we showed for the first time that baseline functional task performance capability can predict the pattern of brain re-organization (increase or decrease in activation in response to treatment).

### Possibility of Two Phases of Neuroplastic Changes in Motor Recovery

In healthy individuals, two phases of skilled motor learning and uniquely associated distinct brain activation patterns were described (Steele and Penhune, 2010); in the first phase of novel task learning, initial task-related brain activation is increased; whereas, when the task is mastered, task-related brain activation becomes more efficient, more focused, that is, decreased in volume of activation (Steele and Penhune, 2010). To date, this complexity of skilled task acquisition has not been well elucidated for stroke survivors, though some have suggested that the initial motor learning phase may be dependent on recruitment of a larger brain volume in order to practice a given task (Feydy et al., 2002; Wittenberg, 2010). The next phase in motor recovery for stroke survivors, consolidation of learning (Steele and Penhune, 2010), may involve the development of more efficient brain control of the movement, which is reflected by more focused task-related brain activity. Those with less baseline impairment may skip the initial phase of motor learning (during which greater volume of activation would have been engaged); rather, they may be able to move directly to the second phase of motor learning, during which they engage a more focused pattern of brain activation. Ability to move immediately to the second, consolidation phase of motor skill acquisition may be due to the existence of sufficient residual perilesional corticospinal pathways. Our results are consistent with the two-phase phenomenon. Those with less impairment at baseline may have already progressed through the initial recovery phase of increased brain activation, and in response to training were developing a more efficient and focused brain activation pattern to re-learn the motor task. Through recognition of the existence of different neuroplastic patterns in neuro-recovery, rehabilitation intervention that involves non-invasive brain stimulation can be designed specifically for different levels of impairment and for different stages of recovery.

### Ipsilesional Motor Regions

It is important to consider the role of the ipsilesional cortex, and specifically perilesional motor-sensory structures with regard to changes in response to treatment. Restoration of perilesional brain function may be the most effective route to complete recovery (Carey et al., 2002; Takahashi et al., 2008; Boyd et al., 2010); however, it may be possible only when there are sufficient existing residual ipsilesional corticospinal motor projections. In fact, better-preserved integrity of the corticospinal tracts, as measured with diffusion tensor imaging (Jang et al., 2005; Qiu et al., 2011) and transcranial magnetic stimulation (TMS; Traversa et al., 2000; Stinear et al., 2012) predicted superior functional outcomes.

Our results for the shoulder/elbow task demonstrated a treatment response in which greater activation of the ipsilesional M1 region correlated with higher gain in motor function (after adjusting for baseline level of motor function; Table 5). Though there is a paucity of information on the shoulder/elbow reach task, we can note, for wrist/hand tasks, the findings have been mixed. For example, several studies described an increase in ipsilesional brain activity in functional recovery of wrist and hand motor tasks (Loubinoux et al., 2003; Takahashi et al., 2008); whereas, others demonstrated a decrease or “focusing” of ipsilesional activation(Schaechter et al., 2002; Ward et al., 2003; Dong et al., 2007). These seemingly conflicting findings, may, in fact, be due to a difference across studies in baseline impairment; this possibility is supported by our findings, in which baseline impairment level determined whether there was an increase or a decrease in ipsilesional brain activation in response to treatment.

### Contralesional Motor-Sensory Regions in Proximal Arm Recovery

In contralesional motor-sensory regions (M1, LPM, S1, and SII), there was treatment-induced, increased activation during shoulder/elbow components of the reach movement which were associated with greater functional improvements, even after adjusting for baseline motor function, age, and time since stroke (Table 5). These findings for a proximal arm task are understandable in light of unique known motor control factors for proximal limb movement. That is, the proximal arm and shoulder muscles used in the reach task receive, to some degree, bi-hemispheric control (Wassermann et al., 1994; Chen et al., 1997; Strutton et al., 2004). Neurophysiological studies using TMS showed that ipsilateral MEPs (i.e., brain stimulation and evoked motor responses on the same side of the body) are more readily obtained for proximal vs. distal upper limb muscles (Bawa et al., 2004). Moreover, in severely impaired individuals, ipsilateral MEPs in the proximal stroke-affected limb muscles are more pronounced than MEPs from contralesional activation of the un-involved limb muscles (Alagona et al., 2001; Schwerin et al., 2008). Therefore, it is likely that the recovery of functional reach is achieved, at least partially, via activation of contralesional corticospinal pathways, i.e., pathways ipsilateral to the stroke-affected limb. These ipsilateral motor control pathways are thought to recruit cortico-reticular-spinal and cortico-tecto-spinal tracts (Ziemann et al., 1999; Schwerin et al., 2008). These results highlight a potential role for the contralesional hemisphere in recovery of the more proximal shoulder/elbow movement components of the reach task.

In studies of the recovery of brain control of wrist/hand or isolated elbow movement, findings have been mixed regarding hemispheric location of recovered brain activation (Wassermann et al., 1994; Chen et al., 1997; Strutton et al., 2004; Whitall et al., 2011). For example, one study reported that motor recovery correlated with increased activation in only ipsilesional activations (Johansen-Berg et al., 2002); but other studies reported change in contralesional activation (a decrease) in response to treatment, which was related to greater gain in distal arm function (Carey et al., 2002; Ward et al., 2003). A third pattern of treatment response was reported for wrist/hand tasks: increased contralesional activation (Schaechter et al., 2002), *n* = 8 (Page et al., 2010); these particular studies were conducted on a smaller scale and without determining a linear relationship with functional improvement (*n* = 4 (Schaechter et al., 2002), *n* = 8 (Page et al., 2010)). Yet, for isolated elbow movement-related activation, following therapy for more mildly impaired subjects, there was an increase in only several bilateral regions; but only the contralesional superior frontal gyrus (that includes SMA) was correlated with improvement in motor function (Whitall et al., 2011).

### Future Directions

Our results provide the rationale to further investigate brain function served by increased activation of the contralesional hemisphere and its potential role in recovery of arm function. In determining the role of the contralesional hemisphere in motor recovery, it will be important in future studies to identify whether activated regions are exhibiting inhibitory or excitatory function. fMRI detects neuroplastic change, yet is unable to elucidate whether the brain activity is excitatory or inhibitory in nature. Other research tools such as repetitive and single pulse TMS and transcranial direct current stimulation (tDCs) have been used to investigate the role of the contralesional motor cortex. Inhibition of the contralesional primary motor area produced temporary improvement in the stroke-affected arm function(Takeuchi et al., 2005; Kirton et al., 2008). Recently, Bradnam et al. (2012) demonstrated in a proof-of-concept study that inhibitory contralesional tDCs had different effects on patients with mild and severe motor deficits; that is, inhibitory tDCs improved motor control in those with mild impairment and worsened motor control in severely impaired individuals. Considering our findings, along with those from brain stimulation studies, we can speculate that the change in extent of the contralesional M1 activity in subjects with mild deficits reflects reduction of transcallosal inhibition. However, for those with severe impairment, it is unclear what type of contralesional neuronal activity (inhibitory or excitatory) is associated with gain in motor function. Furthermore, it is unclear whether other contralesional motor control regions (LPM, S1, SII) may serve a similar function. Studies are needed to further detail whether facilitatory or inhibitory activity in non-primary motor regions governs functional motor gains.

## Conclusion

This study provides new evidence for the complex nature of functional brain changes in response to rehabilitation and recovery of the shoulder/elbow reach task components for those in the chronic stage after stroke. The direction of functional brain change (i.e., increase or decrease in task-related brain recruitment) for recovery of shoulder/elbow motor control depends, in part, on the level of functional impairment at baseline and may represent either different phases or different patterns of neuroplasticity that drive motor function recovery. This bi-directional nature of neuroplastic change following rehabilitation demonstrates a need for an individual approach in neurorehbilitation and different approaches to treatment of distal vs. proximal arm motor control.

Also, the contralesional hemisphere presents as a potentially important player in restoring functional reach; and it is reasonable to consider that the contralesional motor-sensory regions may be important targets for interventions to restore shoulder/elbow function in stroke survivors with varying degrees of motor impairment. It will be important to conduct further studies to address the optimal paradigm for brain training for each hemisphere, for different levels of impairment, and for different muscle groups.

## Conflict of Interest Statement

The authors declare that the research was conducted in the absence of any commercial or financial relationships that could be construed as a potential conflict of interest.

## References

[B1] Alagona G. Delvaux V. Gérard P. De Pasqua V. Pennisi G. Delwaide P. J.. (2001). Ipsilateral motor responses to focal transcranial magnetic stimulation in healthy subjects and acute-stroke patients. Stroke 32, 1304–1309. 10.1161/01.str.32.6.1304 11387491

[B2] Bawa P. Hamm J. D. Dhillon P. Gross P. A. (2004). Bilateral responses of upper limb muscles to transcranial magnetic stimulation in human subjects. Exp. Brain Res. 158, 385–390. 10.1007/s00221-004-2031-x 15316706

[B3] Benjamini Y. Hochberg Y. (1995). Controlling the false discovery rate: a practical and powerful approach to multiple testing. J. R. Stat. Soc. Ser. B 57, 289–300.

[B4] Bogey R. A. Barnes L. A. Perry J. (1992). Computer algorithms to characterize individual subject EMG profiles during gait. Arch. Phys. Med. Rehabil. 73, 835–841. 1514893

[B5] Boyd L. A. Vidoni E. D. Wessel B. D. (2010). Motor learning after stroke: is skill acquisition a prerequisite for contralesional neuroplastic change? Neurosci. Lett. 482, 21–25. 10.1016/j.neulet.2010.06.082 20609381

[B6] Bradnam L. V. Stinear C. M. Barber P. A. Byblow W. D. (2012). Contralesional hemisphere control of the proximal paretic upper limb following stroke. Cereb. Cortex 22, 2662–2671. 10.1093/cercor/bhr344 22139791 PMC4705341

[B7] Butefisch C. Hummelsheim H. Denzler P. Mauritz K. H. (1995). Repetitive training of isolated movements improves the outcome of motor rehabilitation of the centrally paretic hand. J. Neurol. Sci. 130, 59–68. 10.1016/0022-510x(95)00003-k 7650532

[B8] Carey J. R. Kimberley T. J. Lewis S. M. Auerbach E. J. Dorsey L. Rundquist P.. (2002). Analysis of fMRI and finger tracking training in subjects with chronic stroke. Brain 125, 773–788. 10.1093/brain/awf091 11912111

[B9] Carmichael S. T. (2012). Brain excitability in stroke: the yin and yang of stroke progression. Arch. Neurol. 69, 161–167. 10.1001/archneurol.2011.1175 21987395 PMC4698890

[B10] Chen R. Cohen L. G. Hallett M. (1997). Role of the ipsilateral motor cortex in voluntary movement. Can. J. Neurol. Sci. 24, 284–291. 9398974 10.1017/s0317167100032947

[B11] Clarkson A. N. Huang B. S. Macisaac S. E. Mody I. Carmichael S. T. (2010). Reducing excessive GABA-mediated tonic inhibition promotes functional recovery after stroke. Nature 468, 305–309. 10.1038/nature09511 21048709 PMC3058798

[B12] Crafton K. R. Mark A. N. Cramer S. C. (2003). Improved understanding of cortical injury by incorporating measures of functional anatomy. Brain 126, 1650–1659. 10.1093/brain/awg159 12805118

[B13] Daly J. J. Hogan N. Perepezko E. M. Krebs H. I. Rogers J. M. Goyal K. S.. (2005). Response to upper-limb robotics and functional neuromuscular stimulation following stroke. J. Rehabil. Res. Dev. 42, 723–736. 10.1682/JRRD.2005.02.0048 16680610

[B14] Daly J. J. Hrovat K. Pundik S. Sunshine J. Yue G. (2008). fMRI methods for proximal upper limb joint motor testing and identification of undesired mirror movement after stroke. J. Neurosci. Methods 175, 133–142. 10.1016/j.jneumeth.2008.07.025 18786565

[B15] Dean C. M. Shepherd R. B. (1997). Task-related training improves performance of seated reaching tasks after stroke. A randomized controlled trial. Stroke 28, 722–728. 10.1161/01.str.28.4.722 9099186

[B16] Di Pino G. Pellegrino G. Assenza G. Capone F. Ferreri F. Formica D.. (2014). Modulation of brain plasticity in stroke: a novel model for neurorehabilitation. Nat. Rev. Neurol. 10, 597–608. 10.1038/nrneurol.2014.162 25201238

[B17] Dong Y. Dobkin B. H. Cen S. Y. Wu A. D. Winstein C. J. (2006). Motor cortex activation during treatment may predict therapeutic gains in paretic hand function after stroke. Stroke 37, 1552–1555. 10.1161/01.str.0000221281.69373.4e 16645139

[B18] Dong Y. Winstein C. J. Albistegui-DuBois R. Dobkin B. H. (2007). Evolution of FMRI activation in the perilesional primary motor cortex and cerebellum with rehabilitation training-related motor gains after stroke: a pilot study. Neurorehabil. Neural Repair 21, 412–428. 10.1177/1545968306298598 17369516 PMC4067098

[B19] Duncan P. W. Goldstein L. B. Matchar D. Divine G. W. Feussner J. (1992). Measurement of motor recovery after stroke. Outcome assessment and sample size requirements. Stroke 23, 1084–1089. 10.1161/01.str.23.8.1084 1636182

[B20] Elbert T. Pantev C. Wienbruch C. Rockstroh B. Taub E. (1995). Increased cortical representation of the fingers of the left hand in string players. Science 270, 305–307. 10.1126/science.270.5234.305 7569982

[B21] Feydy A. Carlier R. Roby-Brami A. Bussel B. Cazalis F. Pierot L.. (2002). Longitudinal study of motor recovery after stroke: recruitment and focusing of brain activation. Stroke 33, 1610–1617. 10.1161/01.str.0000017100.68294.52 12053000

[B22] Genovese C. R. Lazar N. A. Nichols T. (2002). Thresholding of statistical maps in functional neuroimaging using the false discovery rate. Neuroimage 15, 870–878. 10.1006/nimg.2001.1037 11906227

[B23] Globas C. Lam J. M. Zhang W. Imanbayev A. Hertler B. Becker C.. (2011). Mesencephalic corticospinal atrophy predicts baseline deficit but not response to unilateral or bilateral arm training in chronic stroke. Neurorehabil. Neural Repair 25, 81–87. 10.1177/1545968310382001 20947492

[B24] Hamzei F. Liepert J. Dettmers C. Weiller C. Rijntjes M. (2006). Two different reorganization patterns after rehabilitative therapy: an exploratory study with fMRI and TMS. Neuroimage 31, 710–720. 10.1016/j.neuroimage.2005.12.035 16516499

[B25] Holm S. (1979). A simple sequentially rejective multiple test procedure. Scand. J. Stat. 6, 65–70.

[B26] Jang S. H. Cho S. H. Kim Y. H. Han B. S. Byun W. M. Son S. M.. (2005). Diffusion anisotrophy in the early stages of stroke can predict motor outcome. Restor. Neurol. Neurosci. 23, 11–17. 15846028

[B27] Johansen-Berg H. Dawes H. Guy C. Smith S. M. Wade D. T. Matthews P. M. (2002). Correlation between motor improvements and altered fMRI activity after rehabilitative therapy. Brain 125, 2731–2742. 10.1093/brain/awf282 12429600

[B28] Kirton A. Chen R. Friefeld S. Gunraj C. Pontigon A. M. Deveber G. (2008). Contralesional repetitive transcranial magnetic stimulation for chronic hemiparesis in subcortical paediatric stroke: a randomised trial. Lancet Neurol. 7, 507–513. 10.1016/s1474-4422(08)70096-6 18455961

[B29] Kononen M. Tarkka I. M. Niskanen E. Pihlajamaki M. Mervaala E. Pitkänen K.. (2012). Functional MRI and motor behavioral changes obtained with constraint-induced movement therapy in chronic stroke. Eur. J. Neurol. 19, 578–586. 10.1111/j.1468-1331.2011.03572.x 22040308

[B30] Kopp B. Kunkel A. Flor H. Platz T. Rose U. Mauritz K. H.. (1997). The arm motor ability test: reliability, validity and sensitivity to change of an instrument for assessing disabilities in activities of daily living. Arch. Phys. Med. Rehabil. 78, 615–620. 10.1016/s0003-9993(97)90427-5 9196469

[B31] Lemmens R. J. Timmermans A. A. Janssen-Potten Y. J. Smeets R. J. Seelen H. A. (2012). Valid and reliable instruments for arm-hand assessment at ICF activity level in persons with hemiplegia: a systematic review. BMC Neurol. 12:21. 10.1186/1471-2377-12-21 22498041 PMC3352056

[B32] Levin M. F. Dimov M. (1997). Spatial zones for muscle coactivation and the control of postural stability. Brain Res. 757, 43–59. 10.1016/s0006-8993(97)00204-7 9200498

[B33] Lo A. C. Guarino P. D. Richards L. G. Haselkorn J. K. Wittenberg G. F. Federman D. G.. (2010). Robot-assisted therapy for long-term upper-limb impairment after stroke. N. Engl. J. Med. 362, 1772–1783. 10.1056/NEJMoa0911341 20400552 PMC5592692

[B34] Loubinoux I. Carel C. Pariente J. Dechaumont S. Albucher J. F. Marque P.. (2003). Correlation between cerebral reorganization and motor recovery after subcortical infarcts. Neuroimage 20, 2166–2180. 10.1016/j.neuroimage.2003.08.017 14683720

[B35] Loubinoux I. Dechaumont-Palacin S. Castel-Lacanal E. De Boissezon X. Marque P. Pariente J.. (2007). Prognostic value of FMRI in recovery of hand function in subcortical stroke patients. Cereb. Cortex 17, 2980–2987. 10.1093/cercor/bhm023 17389628

[B36] Luft A. R. McCombe-Waller S. Whitall J. Forrester L. W. Macko R. Sorkin J. D.. (2004). Repetitive bilateral arm training and motor cortex activation in chronic stroke: a randomized controlled trial. JAMA 292, 1853–1861. 10.1001/jama.292.15.1853 15494583 PMC2930817

[B37] McCabe J. Monkiewicz M. Holcomb J. Pundik S. Daly J. J. (2015). Comparison of robotics, functional electrical stimulation and motor learning methods for treatment of persistent upper extremity dysfunction after stroke: a randomized controlled trial. Arch. Phys. Med. Rehabil. 96, 981–990. 10.1016/j.apmr.2014.10.022 25461822

[B38] Nair D. G. Hutchinson S. Fregni F. Alexander M. Pascual-Leone A. Schlaug G. (2007). Imaging correlates of motor recovery from cerebral infarction and their physiological significance in well-recovered patients. Neuroimage 34, 253–263. 10.1016/j.neuroimage.2006.09.010 17070707 PMC2577311

[B39] Nudo R. J. Milliken G. W. Jenkins W. M. Merzenich M. M. (1996a). Use-dependent alterations of movement representations in primary motor cortex of adult squirrel monkeys. J. Neurosci. 16, 785–807. 8551360 10.1523/JNEUROSCI.16-02-00785.1996 PMC6578638

[B40] Nudo R. J. Wise B. M. SiFuentes F. Milliken G. W. (1996b). Neural substrates for the effects of rehabilitative training on motor recovery after ischemic infarct. Science 272, 1791–1794. 10.1126/science.272.5269.1791 8650578

[B41] Page S. J. Harnish S. M. Lamy M. Eliassen J. C. Szaflarski J. P. (2010). Affected arm use and cortical change in stroke patients exhibiting minimal hand movement. Neurorehabil. Neural Repair 24, 195–203. 10.1177/1545968309360501 20107135

[B42] Pascual-Leone A. Torres F. (1993). Plasticity of the sensorimotor cortex representation of the reading finger in Braille readers. Brain 116(Pt. 1), 39–52. 10.1093/brain/116.1.39 8453464

[B43] Picard N. Strick P. L. (1996). Motor areas of the medial wall: a review of their location and functional activation. Cereb. Cortex 6, 342–353. 10.1093/cercor/6.3.342 8670662

[B44] Picard N. Strick P. L. (2001). Imaging the premotor areas. Curr. Opin. Neurobiol. 11, 663–672. 10.1016/s0959-4388(01)00266-5 11741015

[B45] Qiu M. Darling W. G. Morecraft R. J. Ni C. C. Rajendra J. Butler A. J. (2011). White matter integrity is a stronger predictor of motor function than bold response in patients with stroke. Neurorehabil. Neural Repair 25, 275–284. 10.1177/1545968310389183 21357529 PMC3579586

[B46] Rinehart J. K. Singleton R. D. Adair J. C. Sadek J. R. Haaland K. Y. (2009). Arm use after left or right hemiparesis is influenced by hand preference. Stroke 40, 545–550. 10.1161/STROKEAHA.108.528497 19109543

[B47] Sasaki K. Matsunaga T. Tomite T. Yoshikawa T. Shimada Y. (2012). Effect of electrical stimulation therapy on upper extremity functional recovery and cerebral cortical changes in patients with chronic hemiplegia. Biomed. Res. 33, 89–96. 10.2220/biomedres.33.89 22572383

[B48] Schaechter J. D. Kraft E. Hilliard T. S. Dijkhuizen R. M. Benner T. Finklestein S. P.. (2002). Motor recovery and cortical reorganization after constraint-induced movement therapy in stroke patients: a preliminary study. Neurorehabil. Neural Repair 16, 326–338. 10.1177/0888439002016004003 12462764

[B49] Schaechter J. D. Moore C. I. Connell B. D. Rosen B. R. Dijkhuizen R. M. (2006). Structural and functional plasticity in the somatosensory cortex of chronic stroke patients. Brain 129, 2722–2733. 10.1093/brain/awl214 16921177

[B50] Schmidt R. A. (1991). “Motor learning principles for physical therapy,” in Contemporary Management of Motor Control Problems: Proceedings of the II-STEP Conference, ed. Lister M. J. (Alexandria, VA: Foundation for Physical Therapy), 49–67.

[B51] Schwerin S. Dewald J. P. Haztl M. Jovanovich S. Nickeas M. MacKinnon C. (2008). Ipsilateral versus contralateral cortical motor projections to a shoulder adductor in chronic hemiparetic stroke: implications for the expression of arm synergies. Exp. Brain Res. 185, 509–519. 10.1007/s00221-007-1169-8 17989973 PMC2831614

[B52] Shumway-Cook A. Woollacott M. (2007). Motor Control: Translating Research into Clinical Practice. Philadelphia, PA: Linppincott WIlliams and Wilkins.

[B53] Singer R. N. Lidor R. Cauraugh J. H. (1993). To be aware or not aware? What to think about while learning and performing a motor skill. Sport Psychol. 7, 19–30.

[B54] Stark A. Meiner Z. Lefkovitz R. Levin N. (2012). Plasticity in cortical motor upper-limb representation following stroke and rehabilitation: two longitudinal multi-joint FMRI case-studies. Brain Topogr. 25, 205–219. 10.1007/s10548-011-0201-2 21928100

[B55] Steele C. J. Penhune V. B. (2010). Specific increases within global decreases: a functional magnetic resonance imaging investigation of five days of motor sequence learning. J. Neurosci. 30, 8332–8341. 10.1523/JNEUROSCI.5569-09.2010 20554884 PMC6634594

[B56] Stinear C. M. Barber P. A. Petoe M. Anwar S. Byblow W. D. (2012). The PREP algorithm predicts potential for upper limb recovery after stroke. Brain 135, 2527–2535. 10.1093/brain/aws146 22689909

[B57] Strutton P. H. Beith I. D. Theodorou S. Catley M. McGregor A. H. Davey N. J. (2004). Corticospinal activation of internal oblique muscles has a strong ipsilateral component and can be lateralised in man. Exp. Brain Res. 158, 474–479. 10.1007/s00221-004-1939-5 15448962

[B58] Swayne O. B. Rothwell J. C. Ward N. S. Greenwood R. J. (2008). Stages of motor output reorganization after hemispheric stroke suggested by longitudinal studies of cortical physiology. Cereb. Cortex 18, 1909–1922. 10.1093/cercor/bhm218 18234688 PMC2474452

[B59] Takahashi C. D. Der-Yeghiaian L. Le V. Motiwala R. R. Cramer S. C. (2008). Robot-based hand motor therapy after stroke. Brain 131, 425–437. 10.1093/brain/awm311 18156154

[B60] Takeuchi N. Chuma T. Matsuo Y. Watanabe I. Ikoma K. (2005). Repetitive transcranial magnetic stimulation of contralesional primary motor cortex improves hand function after stroke. Stroke 36, 2681–2686. 10.1161/01.str.0000189658.51972.34 16254224

[B61] Teasell R. Mehta S. Pereira S. McIntyre A. Janzen S. Allen L.. (2012). Time to rethink long-term rehabilitation management of stroke patients. Top. Stroke Rehabil. 19, 457–462. 10.1310/tsr1906-457 23192711

[B62] Traversa R. Cicinelli P. Oliveri M. Giuseppina Palmieri M. Filippi M. M. Pasqualetti P.. (2000). Neurophysiological follow-up of motor cortical output in stroke patients. Clin. Neurophysiol. 111, 1695–1703. 10.1016/s1388-2457(00)00373-4 10964084

[B63] Ward N. S. Brown M. M. Thompson A. J. Frackowiak R. S. (2003). Neural correlates of motor recovery after stroke: a longitudinal fMRI study. Brain 126, 2476–2496. 10.1093/brain/awg245 12937084 PMC3717457

[B64] Wassermann E. M. Pascual-Leone A. Hallett M. (1994). Cortical motor representation of the ipsilateral hand and arm. Exp. Brain Res. 100, 121–132. 10.1007/bf00227284 7813640

[B65] Whitall J. Waller S. M. Sorkin J. D. Forrester L. W. Macko R. F. Hanley D. F.. (2011). Bilateral and unilateral arm training improve motor function through differing neuroplastic mechanisms: a single-blinded randomized controlled trial. Neurorehabil. Neural Repair 25, 118–129. 10.1177/1545968310380685 20930212 PMC3548606

[B66] Wittenberg G. F. (2010). Experience, cortical remapping and recovery in brain disease. Neurobiol. Dis. 37, 252–258. 10.1016/j.nbd.2009.09.007 19770044 PMC2818208

[B67] Wittenberg G. F. Chen R. Ishii K. Bushara K. O. Eckloff S. Croarkin E.. (2003). Constraint-induced therapy in stroke: magnetic-stimulation motor maps and cerebral activation. Neurorehabil. Neural Repair 17, 48–57. 10.1177/0888439002250456 12645445

[B68] Wolf S. L. Winstein C. J. Miller J. P. Taub E. Uswatte G. Morris D.. (2006). Effect of constraint-induced movement therapy on upper extremity function 3 to 9 months after stroke: the EXCITE randomized clinical trial. JAMA 296, 2095–2104. 10.1001/jama.296.17.2095 17077374

[B69] Wu C. Y. Huang P. C. Chen Y. T. Lin K. C. Yang H. W. (2013). Effects of mirror therapy on motor and sensory recovery in chronic stroke: a randomized controlled trial. Arch. Phys. Med. Rehabil. 94, 1023–1030. 10.1016/j.apmr.2013.02.007 23419791

[B70] Ziemann U. Ishii K. Borgheresi A. Yaseen Z. Battaglia F. Hallett M.. (1999). Dissociation of the pathways mediating ipsilateral and contralateral motor-evoked potentials in human hand and arm muscles. J. Physiol. 518, 895–906. 10.1111/j.1469-7793.1999.0895p.x 10420023 PMC2269467

